# Supplementary material for: Structural color in Junonia butterflies evolves by tuning scale lamina thickness
Source: eLife. 2020 Apr 7;9:e52187. doi: 10.7554/eLife.52187 (PMC7138606; doi:10.7554/eLife.52187)
Supplement: Transparent reporting form [file elife-52187-transrepform.docx]

***eLife’s* transparent reporting form**

We encourage authors to provide detailed information *within their submission* to facilitate the interpretation and replication of experiments. Authors can upload supporting documentation to indicate the use of appropriate reporting guidelines for health-related research (see [EQUATOR Network](http://www.equator-network.org/%20)), life science research (see the [BioSharing Information Resource](https://biosharing.org/)), or the [ARRIVE guidelines](http://www.plosbiology.org/article/info:doi/10.1371/journal.pbio.1000412) for reporting work involving animal research. Where applicable, authors should refer to any relevant reporting standards documents in this form.

If you have any questions, please consult our Journal Policies and/or contact us: [editorial@elifesciences.org](mailto:editorial@elifesciences.org).

**Sample-size estimation**

- You should state whether an appropriate sample size was computed when the study was being designed
- You should state the statistical method of sample size computation and any required assumptions
- If no explicit power analysis was used, you should describe how you decided what sample (replicate) size (number) to use

Please outline where this information can be found within the submission (e.g., sections or figure legends), or explain why this information doesn’t apply to your submission:

For the number of color forms sampled (i.e. sex and species), we included all members of the genus *Junonia* for which we were able to acquire specimens, with a resulting N=19. Additionally, for a few species we sampled more than one color region per wing, for a final N=23 color regions. This sampling design is shown in Fig. 7A, with all specimens listed in Supplementary File 1.

For spectra and thickness measures, we decided in advance to measure a minimum of 3 scales per specimen/treatment. This was decided in order to meet or exceed typical sample sizes in the existing literature describing pigments and nanostructures in butterfly scales (e.g. doi.org/10.1098/rspb.1999.0794 and PMID: 22323202, 24675561). Sample sizes are reported in the figure legends.

**Replicates**

- You should report how often each experiment was performed
- You should include a definition of biological versus technical replication
- The data obtained should be provided and sufficient information should be provided to indicate the number of independent biological and/or technical replicates
- If you encountered any outliers, you should describe how these were handled
- Criteria for exclusion/inclusion of data should be clearly stated
- High-throughput sequence data should be uploaded before submission, with a private link for reviewers provided (these are available from both GEO and ArrayExpress)

Please outline where this information can be found within the submission (e.g., sections or figure legends), or explain why this information doesn’t apply to your submission:

For the overarching test of this paper, i.e. whether lamina thickness predicts lamina structural color, we used 23 color regions taken from different species/sexes/experimental treatments, which are biologically independent data points. Specimens for many of these species are rare and only a single sample could be acquired for destructive sampling. All specimens used are enumerated with metadata in Supplementary File 1.

For microspectrophotometry, biological replicates are spectra taken from different scales. Each scale is an independent unit of color, and scale color varies dramatically across the wing surface, including between adjacent scales. Technical replicates are spectra taken from the same scale. All spectra results are based on a minimum of 3 biological and 2 technical replicates. This is stated in the Methods and figure legends (Fig. 2,3,5,8,9).

For thickness data taken from micrographs, all results are based on a minimum of 3 scales per treatment/specimen. We also measured different points along individual laminae, for a minimum of 12 total measures per treatment/specimen (stated in figure legend Fig. 8). Because lamina thickness varies extensively within individual scales, measurements taken at different points along a single lamina are not technical replicates. For this reason, we also used tilt calibrations to estimate the range of error in our technique, which are explained in Methods and Fig. 8—Figure supplement B-D and legend.

No data were excluded. Outlier thickness measurements, when they were encountered, were graphed as circles in the boxplot (stated in the legend of Fig. 8).

**Statistical reporting**

- Statistical analysis methods should be described and justified
- Raw data should be presented in figures whenever informative to do so (typically when N per group is less than 10)
- For each experiment, you should identify the statistical tests used, exact values of N, definitions of center, methods of multiple test correction, and dispersion and precision measures (e.g., mean, median, SD, SEM, confidence intervals; and, for the major substantive results, a measure of effect size (e.g., Pearson's r, Cohen's d)
- Report exact p-values wherever possible alongside the summary statistics and 95% confidence intervals. These should be reported for all key questions and not only when the p-value is less than 0.05.

Please outline where this information can be found within the submission (e.g., sections or figure legends), or explain why this information doesn’t apply to your submission:

To test differences in absorption spectra, nonparametric tests were used (Mann-Whitney U, tested separately every 100 nm) because many comparisons showed differences in variance. Figure supplement tables for Fig. 3 and 5 give mean and SD absorbance per specimen per wavelength, as well as U and exact p-value for all of these tests.

Differences in lamina thickness were tested with Type III nested ANOVA, with either treatment (Fig. 1C) or color group (Fig. 8A) as the fixed effect and both individual and scale identity modeled as nested random effects. We used this nested ANOVA model to account for the nonindependence among multiple thickness measurements per scale and multiple scales per individual butterfly. ANOVA was implemented with Satterthwaite’s approximation for uneven sample sizes. For the analysis in Fig. 8A, which is the only case where more than 2 groups were compared in a single test, ANOVA was followed with Tukey’s Honestly Significant Difference test (legend of Fig. 8). Mean, SD, and p values are given in the main text.

Analyses were done in R, and so for all p-values greater than than 2x10^-16^, exact p-values were listed.

(For large datasets, or papers with a very large number of statistical tests, you may upload a single table file with tests, Ns, etc., with reference to sections in the manuscript.)

**Group allocation**

- Indicate how samples were allocated into experimental groups (in the case of clinical studies, please specify allocation to treatment method); if randomization was used, please also state if restricted randomization was applied
- Indicate if masking was used during group allocation, data collection and/or data analysis

Please outline where this information can be found within the submission (e.g., sections or figure legends), or explain why this information doesn’t apply to your submission:

We clustered similar samples into color groups for the analysis in Fig. 8 A-B. Clustering followed the largest natural breaks in the data for two metrics, lamina thickness and weighted average reflectance, which were in good agreement. This is stated in Methods. The unclustered thickness data are also shown in Fig. 8A, and unclustered spectra are shown for a range of samples in Fig. 8 C-G.

**Additional data files (“source data”)**

- We encourage you to upload relevant additional data files, such as numerical data that are represented as a graph in a figure, or as a summary table
- Where provided, these should be in the most useful format, and they can be uploaded as “Source data” files linked to a main figure or table
- Include model definition files including the full list of parameters used
- Include code used for data analysis (e.g., R, MatLab)
- Avoid stating that data files are “available upon request”

Please indicate the figures or tables for which source data files have been provided:

We have provided source data files for Fig. 1, 2, 3, 5, 8, and 9.
